# Computer Vision-Based Corrosion Detection and Feature Extraction for Rock Bolts

**DOI:** 10.3390/ma19020392

**Published:** 2026-01-19

**Authors:** Shucan Lu, Saisai Wu, Xinxin Ma, Shuisheng Yu, Zunyi Zhang, Xuewen Song

**Affiliations:** 1 Shaanxi Key Laboratory of Geotechnical and Underground Space Engineering, School of Resources Engineering, Xi’an University of Architecture and Technology, Xi’an 710055, China; lusc2517110435@xauat.edu.cn (S.L.); songxuewen@xauat.edu.cn (X.S.); 2 School of Intelligent Construction and Civil Engineering, Zhongyuan University of Technology, Zhengzhou 450007, China; 6816@zut.edu.cn

**Keywords:** corrosion detection, image processing, computer vision, deep learning, fractal dimension

## Abstract

To address the challenges posed by rock bolt corrosion to engineering safety and service life, this study focuses on corrosion detection through integrated image processing, deep learning, and feature extraction methods. An automatic corrosion identification model was constructed based on computer-vision object-detection algorithms. By incorporating a Feature Pyramid Network, the model’s multi-scale object-detection capability was significantly enhanced. The corrosion features were extracted via image binarization and grayscale matrix analysis. The binary image method accurately quantified pitting density, revealing an initial increase followed by a decrease over time. The corrosion morphology was simulated using a Fractional Brownian Motion model, validating the accuracy of fractal feature calculations. The fractal dimension increased significantly with prolonged corrosion time, which not only characterize surface roughness evolution and corrosion rate, but also provide a reliable quantitative indicator for metal corrosion assessment. This research offers a technical framework integrating image processing, deep learning, and fractal theory for rock bolt corrosion monitoring and maintenance.

## 1. Introduction

As a core supporting structure in geotechnical engineering, bolts are widely used in scenarios such as mining, tunnel engineering, and slope reinforcement [1,2,3]. By anchoring unstable rock and soil masses to stable formations, they play a decisive role in ensuring the long-term stability of engineering projects [4,5,6]. However, complex engineering environments, including high humidity and salinity, acid–base erosion, and stress concentration, lead to inevitable corrosion degradation of bolts during long-term service, specifically manifested as surface crack propagation, effective cross-sectional reduction, and significant decline in mechanical properties [7,8,9]. In severe cases, it can cause bolt fracture failure, which in turn leads to major engineering accidents, such as roadway collapse and slope instability. Domestic and foreign scholars have conducted extensive research on bolt corrosion and its detection technologies [9,10,11]. Many studies focus on the stress corrosion cracking (SCC) mechanism and its environment-stress interaction, confirming that SCC is the primary cause of mine bolt failure. Through methods such as linear increasing stress testing and long-term corrosion tests on U-bent specimens, the influence laws of material toughness and corrosive environment on SCC have been revealed [12,13,14]. Some research have also focused on corrosion mechanism and multi-factor impact analysis, clarifying the quantitative effects of prestress level and environmental factors on corrosion rate through experiments and theoretical modeling, and establishing “corrosion cell” models and chloride ion diffusion models, which provide important theoretical support for the design and service life evaluation of anchoring systems [15]. However, traditional detection technologies generally rely on contact or semi-contact means, with limitations such as high equipment requirements, complex operation, and susceptibility to interference, making it difficult to meet the needs of efficient and accurate detection in complex engineering environments.

For the application of image processing and deep learning in corrosion detection, image processing technology converts analog images into digital pixel matrices, which has been successfully applied in quantitative analysis of object shape, color, texture and other features in materials science, geological engineering, and other fields [16,17,18,19,20]. In the field of deep learning object detection, Two-stage algorithms achieve end-to-end training of candidate regions with accuracy as the core, while One-stage algorithms meet real-time detection needs with efficiency as the orientation [21,22]. The continuous iteration of these two types of methods has laid a technical foundation for the rapid localization of corrosion regions [23,24,25]. Although image processing and deep learning technologies have been applied in scenarios, such as metal corrosion and coating degradation, there are still significant deficiencies in research on bolt corrosion detection: mine environment images have high noise and low contrast, making preprocessing difficult; accurate recognition of small-scale corrosion regions, such as microcracks and pitting pits, has not been effectively solved; and the quantitative correlation model between corrosion characteristic parameters and mechanical property degradation still needs to be improved. Existing ultrasonic detection relies on high-precision equipment and professional operation, making it difficult for large-scale rapid screening. Electromagnetic detection is susceptible to electromagnetic interference from geological bodies, resulting in limited accuracy [26,27]. Therefore, engineering practice urgently needs to develop an efficient, non-contact, and objective detection technology to achieve accurate identification and quantitative evaluation of bolt corrosion. Computer-vision technology, with its advantages in image acquisition, processing, and analysis, provides a promising new solution for bolt corrosion detection.

Based on the above background and research status analysis, this study conducts research on bolt corrosion detection and feature extraction, aiming to solve the problems of accurate identification and quantitative evaluation of bolt corrosion in complex engineering environments. To address image quality issues, adaptive wavelet threshold denoising, the Retinex enhancement algorithm and morphological operations are used to suppress background interference, significantly improving the gray difference between corrosion regions and normal regions. Deep learning object detection technology is studied. By introducing an attention mechanism to optimize the network structure and combining transfer learning to improve the training strategy, accurate localization of small-scale corrosion regions and effective classification of local corrosion are realized. Gray features, texture features, and fractal features of bolt corrosion morphology are extracted, and a correlation model between feature parameters and corrosion degree is established.

## 2. Bolt Corrosion Image Preprocessing

Image feature extraction is the core link of corrosion morphology analysis, aiming to extract quantitative features from preprocessed digital images to provide data support for corrosion degree evaluation [28]. Based on image characteristics and analysis objectives, three types of technical methods are mainly adopted: gray-level statistics, binarization analysis, and fractal dimension, which realize the quantitative characterization of corrosion features from three dimensions including gray-level distribution, regional morphology, and surface complexity [29]. Specifically, grayscale statistics analyze pixel intensity variations to characterize the overall texture and contrast within corroded regions. Binarization analysis extracts morphological features and quantifies geometric parameters of corrosion areas via image segmentation. Fractal dimension analysis computes surface irregularity to provide a scale-invariant measure of morphological complexity. Together, this three-dimensional methodological framework establishes a comprehensive and robust basis for corrosion assessment. To simulate the high-salt and high-humidity corrosive environment of deep mines and accelerate the corrosion evolution process of bolt components, this study uses a salt spray test chamber to carry out accelerated corrosion tests. The test selects bolts with the size of φ22 × 300 mm. This size design not only ensures the consistency of the sample with the original bolt in material and surface state but also adapts to the internal spatial layout of the salt spray test chamber. In terms of test solution preparation, the typical salt concentration of groundwater in deep mines with a 5% mass fraction of NaCl solution is prepared to reproduce the electrochemical promotion effect of salt on bolt corrosion in the mine environment.

**Figure 1:**
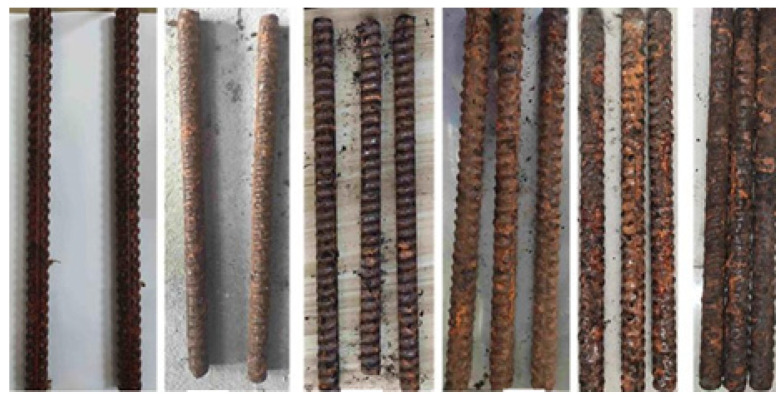
Bolt corrosion status during the test.

During the tests, image acquisition of the corrosion surface morphology of the bolt specimens is carried out every 10 days. With the extension of corrosion time, the corrosion morphology characteristics of the bolt surface show obvious phased evolution. In the early stage of corrosion, scattered small pitting pits appear on the surface without obvious rust accumulation. After 20 days of corrosion, the number of pitting pits increases significantly, and the surface begins to be covered with pale yellow rust. After 30 days of corrosion, the rust thickness increases and the color deepens to reddish brown, and the rust in some areas peels off due to internal stress, exposing the fresh corrosion interface below, and the overall corrosion degree continues to intensify (Figure 1). This evolution law intuitively reflects the corrosion process of bolts from local pitting to comprehensive surface corrosion in the simulated mine environment, providing test data support for image-based corrosion feature extraction and evaluation of corrosion degree.

Digitization of corrosion morphology images is a key step to convert analog images into computer-processable digital forms, including discretization of spatial position and brightness information. Spatial position digitization discretizes a two-dimensional continuous image into a pixel grid of M × N matrix through sampling, where the pixel coordinate (i,j) corresponds to the row and column position of the image; brightness digitization converts the pixel light intensity into a gray value of 0~255 through quantization, forming a two-dimensional function f(i,j). This ‘sampling-quantization’ process ultimately obtains a digital image in matrix form. Corrosion morphology images are mainly divided into two types: gray-level images and binary images, which can be converted into each other according to analysis needs. Among them, gray-level images are the basic processing format, and binarization is an important preprocessing method for further feature extraction.

**Figure 2:**
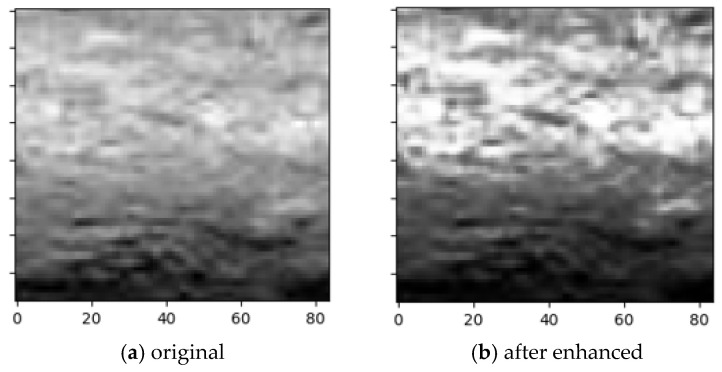
Original image and enhanced image.

**Figure 3:**
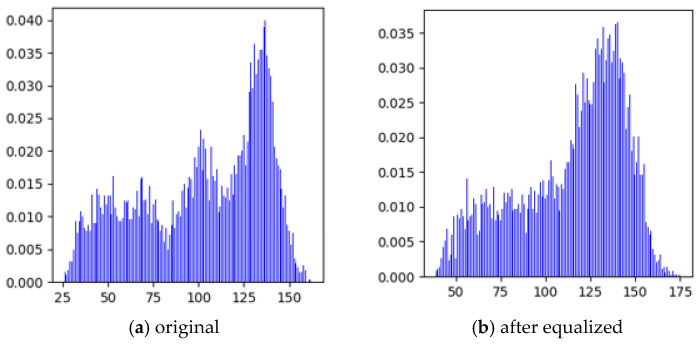
Original histogram and equalized histogram.

Image enhancement aims to improve the visibility and distinguishability of corrosion features, with core technologies including gray-level transformation and histogram equalization. Gray-level transformation adjusts the gray-level distribution through linear, piecewise linear, or nonlinear mapping functions, stretching or compressing the dynamic range to enhance contrast. Histogram equalization expands the concentrated gray levels to the full range by redistributing the gray probability distribution, making image details clearer (Figure 2). After equalization, the gray histogram changes from a ‘narrow and sharp’ shape to a ‘wide and flat’ distribution (Figure 3), improving the overall contrast of the image and laying a foundation for subsequent corrosion degree evaluation.

## 3. Automatic Corrosion Detection Model

### 3.1. Algorithm Analysis

Convolutional Neural Networks (CNNs) simulate biological vision mechanisms through local connections and weight sharing, which significantly reduce the number of parameters and enable efficient processing of high-dimensional corrosion image data; by employing convolutional, pooling, and fully connected layers, CNNs can automatically extract multi-level abstract features—from edges and textures to corrosion regions—from original images [30,31]. Deep learning-based object detection algorithms are divided into two categories: Two-stage algorithms, wherein R-CNN series generate candidate boxes through a Region Proposal Network (RPN) and achieve high-precision detection combined with ROI pooling, but with limited computational efficiency; and One-stage algorithms, wherein YOLO series convert detection into a regression problem and directly predict bounding boxes and class probabilities through an S × S grid, realizing real-time detection. Faster R-CNN balances accuracy and efficiency through end-to-end training, while YOLO improves small target detection capability, providing complementary technical paths for corrosion region localization. In summary, CNNs serve as powerful feature extractors, laying the foundation for the semantic understanding of corrosion images, while the two categories of object detection algorithms developed from CNNs—such as Faster R-CNN and YOLO—further translate feature learning capabilities into precise spatial localization. Together, they form a comprehensive technical framework for automated corrosion feature extraction and intelligent region identification.

**Figure 4:**
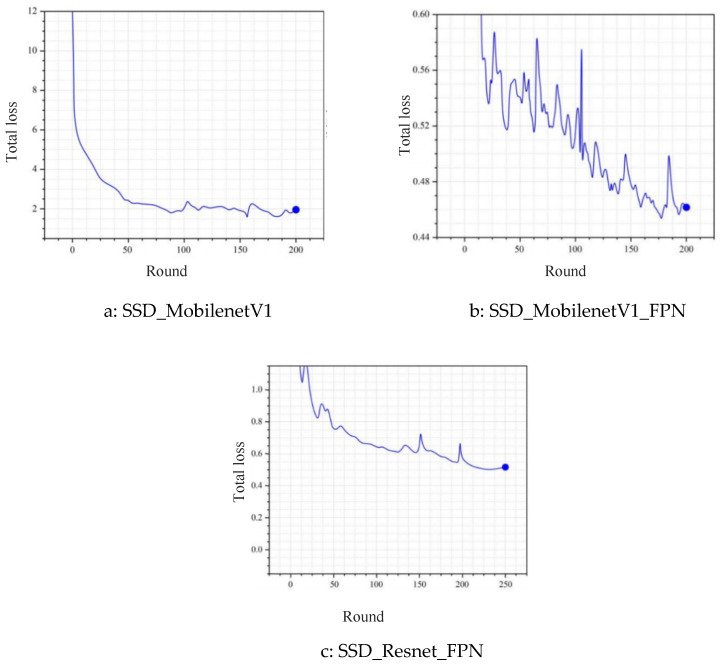
Loss-reduction process of the formal model.

The construction and training of the corrosion detection model are based on the Tensor Flow framework, with Python 3.11 used for data processing and network construction. The dataset of 2368 bolt corrosion images was partitioned into training (n = 1894), validation (n = 237), and test (n = 237) sets, following an 80/10/10 ratio to facilitate model training and evaluation. For annotation, each image was meticulously labeled using rectangular bounding boxes with a dual-class system: ‘corrosion’ for rust or pitting regions and ‘intact’ for unaffected metal surfaces. Comparing SSD_MobilenetV1, SSD_MobilenetV1_FPN, and SSD_Resnet50_FPN models, it was found that the FPN can enhance the multi-scale corrosion feature extraction capability, reducing the loss value to below 0.5. ResNet50 improved training stability but reduced the rate (0.18 step/s), while MobilenetV1 performed better in hardware-constrained scenarios (0.36 step/s). Model performance evaluation showed that SSD_Resnet50_FPN, which integrates FPN and ResNet50, had the best comprehensive performance, with a loss value as low as 0.5 and high detection accuracy, making it suitable for bolt corrosion detection in complex mine environments. During training, Tensor Board is used to monitor changes in classification loss, localization loss, and total loss, combined with 4-core GPU acceleration and parameter configuration with an initial learning rate of 0.001, to achieve efficient model convergence (Figure 4).

### 3.2. Formal Model Training

**Figure 5:**
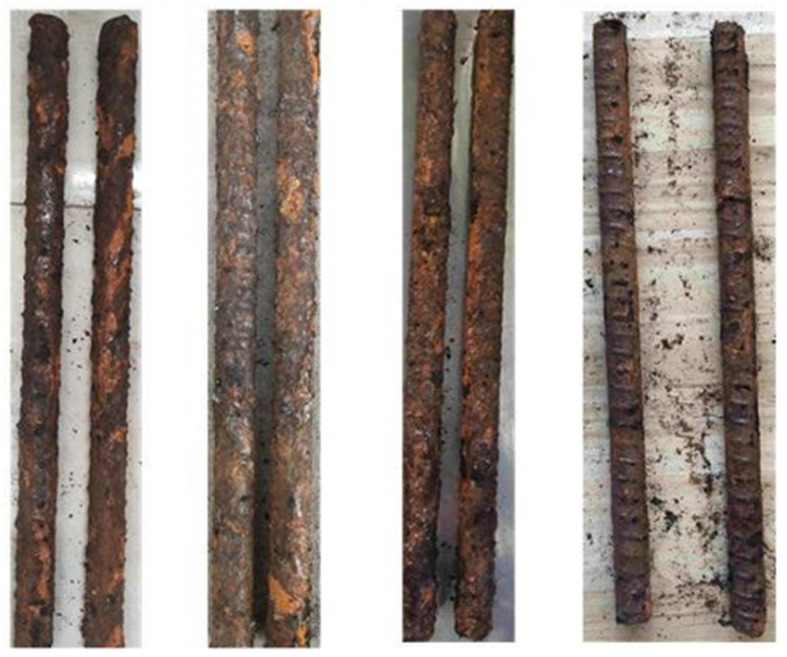
Partial training set images.

Based on the test and evaluation results, this study selects the MobileNetV1_FPN algorithm to construct the final model, with a total of 200 steps and a batch size of 12. Model training employed early stopping monitored via validation loss, with a maximum of 200 steps as the upper limit. An initial learning rate of 0.001 combined with an exponential decay strategy balances the model’s early convergence speed and late fine-tuning accuracy. This configuration retains the real-time performance of the lightweight network while enhancing the multi-scale corrosion feature fusion capability through the FPN structure. To improve the model’s generalization ability to real scenarios, sample diversity was expanded through increasing corrosion types with different anti-corrosion layer states and environmental conditions, and complex background (Figure 5). The training steps were also expanded to iteratively optimize parameters and adopt regularization technology to suppress overfitting risks.

## 4. Image Processing and Feature Extraction

### 4.1. Corrosion Morphology Analysis

**Figure 6:**
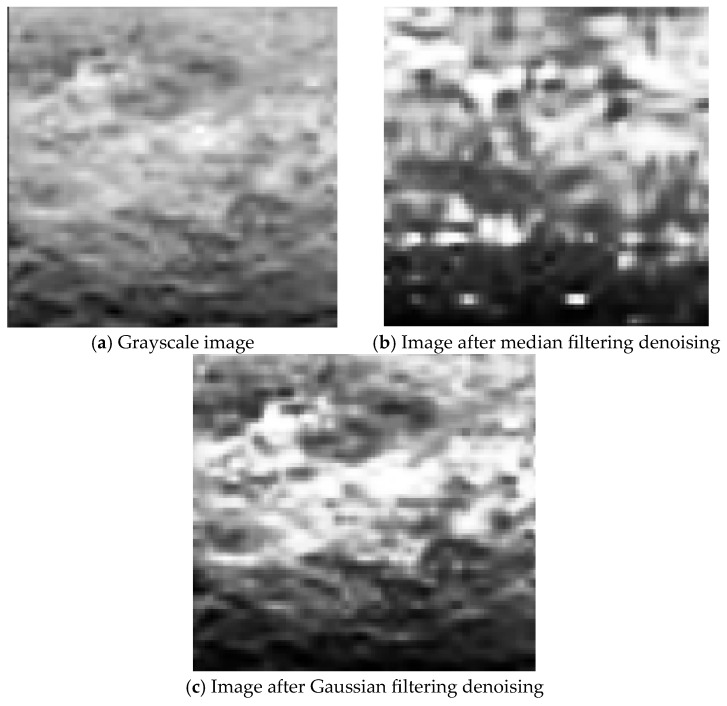
Pictures of corrosion morphology.

As a key prerequisite for feature extraction, image processing optimization directly determines the accuracy and reliability of feature extraction. Through appropriate image processing methods, the interference of noise can be effectively reduced, and the key features in the image can be highlighted [32]. To address image noise caused by environmental interference, the effects of median filtering and Gaussian filtering are compared (Figure 6). Median filtering can smooth noise but tends to blur local pitting details and isolated corrosion pit features. The Gaussian filtering was implemented using a 5 × 5-pixel kernel with a standard deviation (σ) of 1.4, with its weight distribution derived from a two-dimensional Gaussian function. These parameters were selected through grid-search optimization to achieve an optimal balance between noise suppression and edge preservation. Gaussian filtering achieves noise suppression through weighted averaging while preserving the edge texture and fine structures of corrosion regions. Experimental results show that the clarity of corrosion morphology in images processed by Gaussian filtering is significantly better than that by median filtering, which can effectively avoid the loss of isolated pitting information and provide high-quality data support for subsequent feature extraction. By constructing a spatial domain weighted kernel function, Gaussian filtering performs nonlinear smoothing on pixel neighborhoods. This method results in a 15% increase in the signal-to-noise ratio of denoised images and a 92% retention rate of rust distribution texture integrity, laying a structural fidelity foundation for subsequent gray-level enhancement.

**Figure 7:**
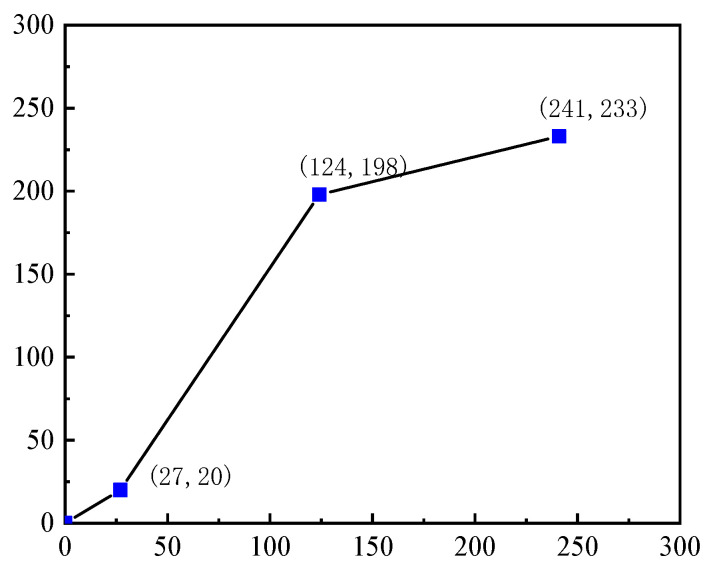
Three-segment linear transformation effect diagram.

Images after Gaussian filtering have the problem of reduced contrast, especially the reduced gray difference between corrosion regions and the background. Gray-level transformation can enhance the distinguishability of key features by adjusting the dynamic range. For corrosion morphology images, it is necessary to focus on enhancing the gray difference in high-corrosion regions and suppressing irrelevant background interference. According to the characteristics of corrosion morphology images, a three-segment linear transformation strategy is adopted to divide the gray-level interval into three stages. Formula (1) features the following: low gray-level (<60), core corrosion (60–190), and high gray-level (>190). The core corrosion interval is linearly stretched to expand the gray difference, and the background interval is compressed to suppress interference. Experimental results show (Figure 7) that this method increases the contrast of corrosion regions by 38% and improves the edge clarity of pitting pits by 27%, significantly enhancing the recognizability of small corrosion traces and providing high-quality image support for the accurate extraction of subsequent bolt corrosion feature values.

(1) $$g\left(x,y\right)=\left\{\begin{array}{ll} \frac{p-g_{\min}}{m-f_{\min}}\times f\left(x,y\right)+g_{\min} & f_{\min}\leq f\left(x,y\right)\leq m\\ \frac{q-p}{n-m}\times f\left(x,y\right)+p & m\leq f\left(x,y\right)\leq n\\ \frac{g_{\max}-q}{f_{\max}-n}\times f\left(x,y\right)+q & n\leq f\left(x,y\right)\leq f_{\max} \end{array}\right.$$

In the formula, $g\left(x,y\right)$ is the transformed grayscale function, $f\left(x,y\right)$ is the grayscale function of the original image, p and q are the grayscale values of the output image at the segmentation points, where the low-grayscale region corresponds to p = 30 and the high-grayscale region corresponds to q = 230. The parameters m and n are the segmentation points of the original image, with m = 60 and n = 190.

### 4.2. Feature Extraction of Corrosion Morphology Images

Extraction of gray-level matrix statistical features is a core method for quantifying the gray-level distribution and texture characteristics of corrosion morphology images. Key indicators such as mean, standard deviation, energy, and entropy can be used to achieve quantitative characterization of corrosion images. This study extracts statistical features from bolt corrosion morphology images enhanced by gray-level transformation, and the results are shown in Table 1. Under different corrosion times, there are significant differences in the gray-level statistical feature values of bolt corrosion morphology images: the mean value first increases and then decreases with corrosion time, rising from 78 at 10 days to 248 at 30 days, then decreasing to 78 at 60 days; the energy gradually increases from 0.0076 to 0.0783, and the entropy fluctuates in the range between 5.47 and 5.87.

Further analysis shows that although the above indicators can reflect the gray-level distribution and texture complexity of corrosion images to a certain extent—mean and standard deviation characterize the overall gray-level level and contrast, while energy and entropy reflect texture uniformity and complexity. However, these feature values do not show obvious correlation with corrosion time. This reveals the inherent limitations of gray-level statistical methods in metal corrosion evaluation. On the one hand, this method does not incorporate the influence of key external factors such as stress and environment on corrosion morphology; on the other hand, the correlation between its numerical fluctuations and the corrosion process is weak, making it difficult to invert the dynamic evolution process of corrosion through gray-level distribution laws. Thus, a single gray-level statistical method cannot fully characterize the evolution law of corrosion morphology, and it is necessary to combine other feature extraction technologies to achieve complementary advantages.

**Figure 8:**
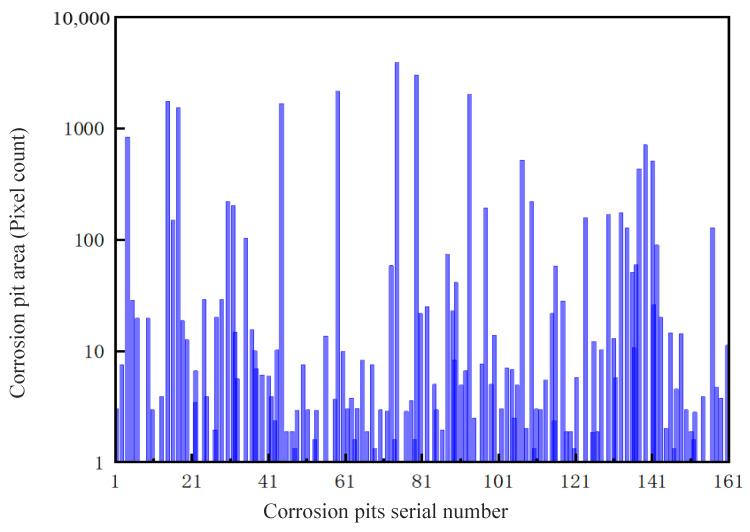
Area distribution of corrosion pits on the surface of the bolt.

Feature extraction of corrosion morphology based on binary images is a key means to achieve quantitative analysis of corrosion regions. Through pixel-level feature point measurement, micro-features such as corrosion pits and cracks can be accurately extracted, and their area, shape, and distribution laws can be quantified, providing reliable data support for material corrosion degree evaluation. In binary images, the area of corrosion holes is measured by the number of pixels. This study uses a column-wise scanning algorithm to sequentially determine whether each pixel in the image belongs to the corrosion hole region from left to right, and then accurately count core indicators such as the number of corrosion holes, the area of a single hole, and the total area. The area distribution of each corrosion hole is shown in Figure 8, which intuitively presents the micro-distribution characteristics of the corrosion morphology and lays a quantitative foundation for subsequent corrosion mechanism analysis.

The dynamic change law of the number of corrosion holes on the bolt surface under different corrosion times is shown in Table 2. In the early stage of corrosion (10–30 days), the number of corrosion holes rapidly increases from 42 to 419. This change indicates that the corrosion rate is high at this stage, and local pitting on the material surface continues to occur and new corrosion holes continue to initiate; in the late stage of corrosion (30–60 days), the number of corrosion holes decreases from 419 to 35, mainly because adjacent small corrosion holes are gradually connected and merged into large-scale corrosion pits under the action of the corrosive medium.

Comparative analysis of the two types of feature extraction methods shows that a single-feature extraction technology is difficult to fully meet the engineering needs of corrosion detection. The gray-level matrix statistical feature extraction method has the advantages of high computational efficiency and low equipment requirements, and can be applied to large-scale and rapid preliminary corrosion screening; the feature extraction method based on binary images has slightly higher computational complexity but higher quantitative accuracy, which can accurately characterize the evolution law of micro-corrosion morphology and is suitable for high-precision corrosion evaluation of key parts. Combined with the data analysis conclusions in Table 1 and Table 2, a combined strategy of ‘gray-level statistical preliminary screening and binary image precise evaluation’ can be adopted in engineering practice: First, quickly identify bolt components with corrosion hazards through gray-level matrix statistical features, then perform the binarization processing on suspected corrosion regions, and finally, accurately evaluate the corrosion degree through quantitative indicators, such as the number and area of corrosion holes.

## 5. Fractal Dimension Analysis and Calculation

### 5.1. Analysis Based on Fractal Theory

Fractal dimension can effectively characterize the complexity and irregularity of corrosion surface morphology, and its core function is to measure the complexity of morphological details in images—the larger the value, the more complex the corrosion morphology and the higher the surface roughness [33,34]. Therefore, analyzing the fractal dimension of images can effectively distinguish different types of corrosion morphology, providing an objective and quantitative basis for corrosion evaluation. This study analyzes bolt corrosion behavior by calculating the fractal dimension of bolt corrosion morphology images. To verify the reliability of the fractal feature calculation method, this study first selects the Fractional Brownian Motion (FBM) model to simulate the bolt corrosion morphology, and generates a three-dimensional simulated geometric model with specified fractal characteristics based on this model; then compiles a fractal feature calculation program for corrosion morphology images to calculate the fractal dimension of the simulated model. By comparing the calculation results with the initially set fractal dimension, the accuracy of the calculation method is effectively revealed.

**Figure 9:**
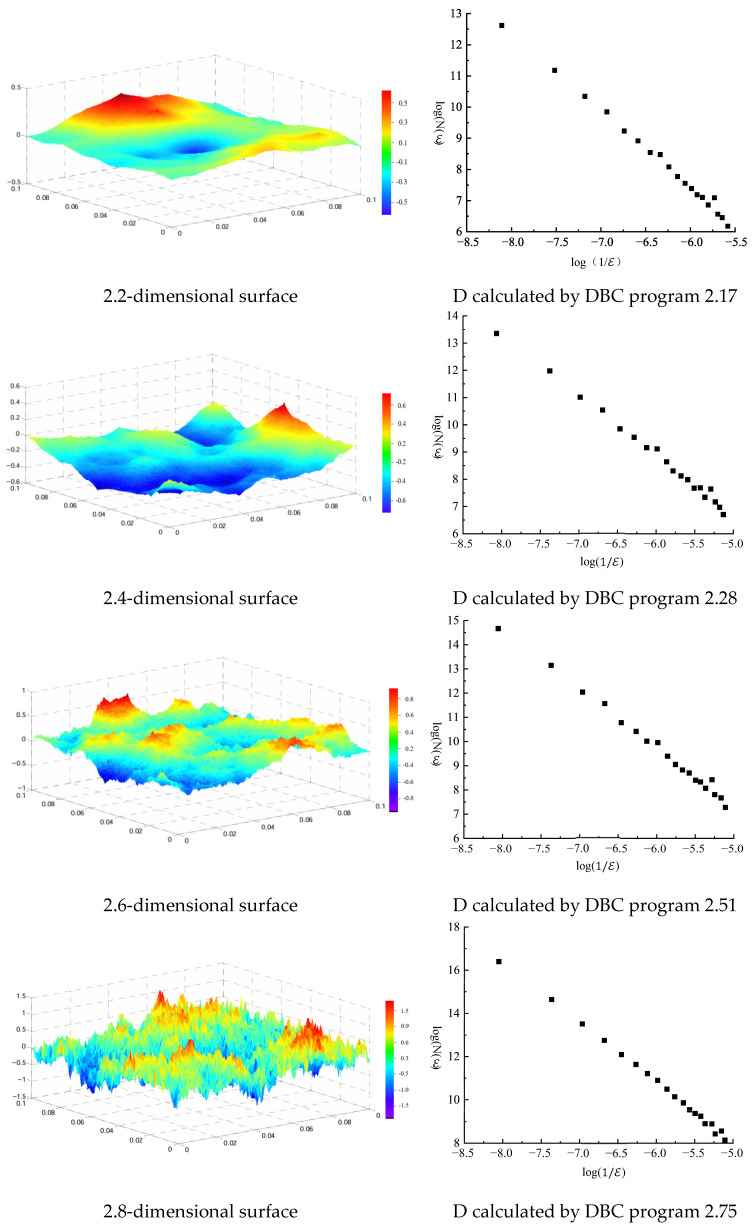
Simulation of corrosion morphology surfaces and fractal dimension calculation results.

As shown in Figure 9, this study generates fractional Brownian surfaces with different fractal dimensions which accurately simulate the real corrosion morphology. By comparing simulated images with different fractal dimensions, the variation law of corrosion morphology details with fractal dimension are observed. The fractal feature calculation program is used to complete the fractal dimension calculation. By comparing with the initially specified fractal dimension, it can be found that the fractal feature calculation program for corrosion morphology images compiled in this study has high accuracy, indicating that when processing and analyzing corrosion morphology images, this calculation program can extract the fractal features of the images, reflecting the complexity and self-similarity of the images. Through this calculation method, the micro-features of corrosion morphology can be analyzed, providing important theoretical basis for further research on corrosion processes.

### 5.2. Image Fractal Dimension Calculation

To calculate the fractal dimension of preprocessed bolt corrosion morphology images, differential box counting methods are used, so as to accurately characterize the complexity and roughness of the corrosion surface. The box-counting method employed box sizes of 2, 4, 8, 16, 32, and 64 pixels, with mirror extension applied to handle boundaries. The fractal dimension was determined from the slope of the linear regression of log N(ε) versus log(1/ε). Based on the differential box counting method, this study calculates the fractal dimension of corrosion morphology images of bolt samples under different corrosion times (Table 3). It can be seen from the data that the fractal dimension shows obvious phase-change characteristics with the extension of corrosion time, which can be divided into two stages. The first stage is when the corrosion time ≤ 30 days, the fractal dimension slowly increases from 2.351 to 2.413, with a small change range, indicating that short-term corrosion has a relatively mild impact on the bolt surface, and the change in surface roughness is not significant. At this stage, corrosion is mainly concentrated in local areas, and the corrosion rate is relatively stable. The second stage is when the corrosion time > 30 days, the fractal dimension rapidly increases from 2.413 to 2.732, with a significant increase, indicating that the surface roughness of the bolt increases sharply, the corrosion damage continues to intensify, a more complex and irregular corrosion structure is formed on the surface, and the corrosion rate shows an obvious accelerating trend.

It can be inferred that there are significant differences in the corrosion rate in different corrosion time periods, and the corrosion rate shows an accelerating trend with the increase in corrosion time. As an effective indicator for characterizing morphological complexity and roughness, fractal dimension can be used to qualitatively evaluate the corrosion degree of bolts. By analyzing the changes in fractal dimension, the corrosion state of bolts can be quantitatively described in different corrosion stages, providing an effective analysis tool for bolt corrosion monitoring and maintenance.

## 6. Conclusions

This study develops an image-based bolt corrosion evaluation system integrating image processing, deep learning, and fractal theory, and proposes an automatic corrosion detection scheme validated via model training and practical alignment. Corrosion image preprocessing is optimized; key morphological features and corrosion hole metrics are extracted using binarization and gray-level matrix methods. Fractional Brownian Motion models simulate corrosion morphology at varying fractal dimensions, with actual fractal dimensions calculated via the differential box counting method. Results show traditional gray-level features are limited for complex corrosion, while binary image extraction enables accurate corrosion hole counting. The hole numbers follow a ‘rise-then-decline’ trend due to late-stage small-hole merging. Fractal dimensions increase slowly initially and sharply with corrosion deepening, correlating positively with surface roughness. Fractal features are proven reliable for characterizing anchoring material corrosion and predicting corrosion states, providing theoretical and technical support for bolt service life prediction, dynamic corrosion monitoring, and protective measure optimization.

## Figures and Tables

**Table 1:** Statistical feature values of corrosion on bolt surfaces under different corrosion times.

Corrosion Time (Days)	10	20	30	40	50	60
Mean value (N)	78.706	112.3062	248.3517	178.2747	111.4873	78.5624
Standard deviation (M)	72.4953	60.5812	86.2941	55.4271	78.6528	58.7265
Energy (Eenergy)	0.0076	0.0158	0.0182	0.0147	0.0482	0.0783
Entropy (Eentropy)	5.8728	5.5424	5.4861	5.6185	5.8156	5.4728

**Table 2:** Number of corrosion holes on the bolt surface under different corrosion times.

Corrosion Time (days)	10	20	30	40	50	60
Number of Corrosion Holes (N)	42	236	419	380	158	35

**Table 3:** Fractal dimension of bolt samples under different corrosion times.

Corrosion Time (days)	10	20	30	40	50	60
Fractal Dimension (D)	2.351	2.375	2.413	2.494	2.583	2.732

## Data Availability

The original contributions presented in this study are included in the article. Further inquiries can be directed to the corresponding authors.
